# Context Stability in Habit Building Increases Automaticity and Goal Attainment

**DOI:** 10.3389/fpsyg.2022.883795

**Published:** 2022-06-10

**Authors:** Marco Stojanovic, Axel Grund, Stefan Fries

**Affiliations:** ^1^Department of Psychology, Bielefeld University, Bielefeld, Germany; ^2^Luxembourg Centre for Educational Testing – LUCET, Faculty of Humanities, Education and Social Sciences, University of Luxembourg, Luxembourg, Luxembourg

**Keywords:** habit formation, longitudinal study, context stability, goal attainment, automaticity, app intervention

## Abstract

In this paper, we investigate the effects of context stability on automaticity and goal attainment in intentional habit building. We used hierarchical growth curve modeling and multilevel mediation to test our hypotheses on two datasets. In Study 1, *N* = 95 university students (*N* = 2,482 habit repetitions) built new study habits over a period of 6 weeks with manipulated context stability. One group was instructed to constantly vary the context of their habit repetitions by changing rooms and times and the other group was instructed to keep the context of habit performance stable. In Study 2, *N* = 308 habits (*N* = 2,368 habit repetitions) from *N* = 218 users of a published habit building app were analyzed without manipulating but measuring context stability. We found the same pattern in both datasets: Context stability predicted more automaticity and higher habit repetition goal attainment. We also found that the effect of context stability on habit repetition goal attainment was partially mediated by automaticity in both datasets. These results show that context does not only act as a trigger for habit instigation but also has an ongoing effect on habit execution.

## Introduction

You may have expected a quote by some famous person here, but you find a small demonstration of how something in the wrong context can draw attentional resources, cause surprise or even trigger mental friction.

-*Marco Stojanovic*

We cannot escape context. Everything that is, exists in a certain context. Be it a thought, a word, or a behavior. The human brain evolved to adapt to frequently encountered contexts by automatically activating behavioral responses that yielded beneficial results in the past (Wood and Rünger, 2016). We often only recognize how automatic a behavior has become after discovering how odd it feels when trying to perform it in another environment or by experiencing the unintended execution of a behavior triggered by a context in which it was usually performed—so-called action slips. Moving an automized behavior from its accustomed context to a new one usually leads to interference in the behavioral execution. Musicians often experience music performance anxiety when switching from the comfortable training environment to an environment in which they are evaluated by others (Patston, 2014), and Facebook users—especially those who posted frequently in the past—posted less after the design of the platform had been changed (Anderson and Wood, 2021). On the other hand, performing a less automized behavior can lead to slipping into an unintended, more automatic behavior by faulty activation of schemas strongly tied to the current context (Norman, 1981). Think of trying to make a healthy morning smoothie in the kitchen for the first time but mindlessly reaching for the good old coffee mug. Behavior and context are deeply intertwined.

Context, frequency, and automaticity are central concepts in modern habit research. Classic, simplistic definitions of habit equaled frequency of behavior with habit (Triandis, 1977). Why is something done frequently? Because it is a habit. Why is it a habit? Because it is done frequently. To resolve this tautology and to distinguish habits from deliberate behavior, automaticity became a new, central variable: A behavior is performed frequently, because it is automatically triggered and performed (Verplanken and Orbell, 2003; Gardner, 2012). But why is a habit initiated in the first place? This is where the third key element of habit comes into play: Context. Habit-specific contexts trigger habit execution (Wood and Rünger, 2016). Integrating all of these concepts, Verplanken (2018, p. 4) defines habits as “(…) memory-based propensities to respond automatically to specific cues, which are acquired by the repetition of cue-specific behaviours in stable contexts.”

In past habit research, context stability was inferred through instructions to keep it stable (e.g., Lally et al., 2010), multiplied with frequency to obtain a habit measure (Galla and Duckworth, 2015) or defined by location (e.g., Verplanken et al., 2008). The great majority of the habit studies have a cross-sectional design and typically conceptualize context as a mere cue triggering habits and not as a factor with an ongoing influence on behavioral execution. Longitudinal studies, in which the intentional acquisition of habits is tracked within-person after each habit repetition, are scarce. We identified only five relevant published papers to date (Lally et al., 2010; Fournier et al., 2017; Stojanovic et al., 2020, 2021; Van der Weiden et al., 2020) and in none of them context stability has been tested for a possible influence on the habit building process. To fill this gap and add to the scarce longitudinal habit research, we measure perceived context stability in two longitudinal studies (including a context stability manipulation in Study 1), and test the effects of context stability during habit acquisition on automaticity and goal attainment, which are both variables tied to the quality of habit execution.

### Context Stability Supports Behavioral Automatization

When investigating habit formation, automaticity is typically the dependent variable, which grows over time by habit repetition (Lally et al., 2010; Stojanovic et al., 2020, 2021). A very automized habit would be considered a strong habit. In a habit, there are two spots for automatization: The connection between the context cue and the beginning of the habit (habit instigation) and the performance of the more or less automatic behavioral sequence following the contextual trigger (habit execution; Gardner et al., 2016). Empiric support for the popular notion of “the most important thing is to start” is provided by Gardner et al. (2016) who found that habitual instigation automaticity is more predictive for behavioral frequency than habit execution automaticity. Non-specific automaticity on the other hand reduces motivational interference (i.e., bad mood, distractibility, thoughts about alternatives, task switching and low persistence) during habit performance (Stojanovic et al., 2020, 2021).

We define context as the product of the physical environment and the point in the flow of the day. Habits need a time and a space. There is a whole research field investigating the effects of time on the human physiology and behavior: Chronobiology. The primary time cue (*zeitgeber*) is light exposure, which influences melatonin levels and thus the circadian rhythm (e.g., Khalsa et al., 2003). But also food intake (Lewis et al., 2020) and exercise (Lewis et al., 2018) can act as zeitgebers influencing the functionality of the circadian system. Our neurophysiology models time as a function of external cues. Concerning the space aspect of our context definition, the influence is even more direct. Noise can lead to distraction (Vasilev et al., 2018), external temperature influences human thermoregulation (van Marken Lichtenbelt et al., 2007), and being watched while doing a difficult task leads to rising blood pressure (Gendolla and Richter, 2006). Whether we notice it or not, our whole body reacts and adapts to the physical environment as well as the time in the flow of a day.

Traditionally, in habit research, context is seen as a trigger initiating a habit (Wood and Rünger, 2016). Ouellette and Wood (1998) showed in a meta-analysis that behavior that was previously performed in stable (vs. unstable) contexts was considerably less guided by intention. Correspondingly, changing contexts (i.e., removing triggers) leads to less habitual behavior. Studies in the field of habit discontinuity research show that students’ exercise, newspaper reading, and TV habits can be disrupted after switching universities (Wood et al., 2005), environmentally concerned people could lower their habitual car use after moving from home (albeit slowly falling back into their old habits over time while adapting to the new environment; Thomas et al., 2016), and physical activity habits were disrupted by the COVID-19 lockdowns in France and Switzerland in the spring of 2020 (Maltagliati et al., 2021). So, typically, context is conceptualized as a cue triggering a habit, or an instigation cue as Gardner et al. (2016) would put it. However, in this research we want to show that the context does not only have an influence on triggering a habit, but also on the performance of the behavioral chain after the instigation, or the habit execution as Gardner et al. (2016) would put it. The contextual influence lingers.

Context affects performance. Next to the in the introduction mentioned performance anxiety effect of musicians after switching from the training context to an evaluative context (Patston, 2014), students’ performance in proctored exams seem also to be influenced by context as it is higher when taken on-site—in the learning environment—rather than remote (Wuthisatian, 2020). In their meta-analysis, Smith and Vela (2001) found support for reliable reinstatement effects, meaning that memory is enhanced when the learning context is reinstated at recall. Chess experts have built a highly automized associative knowledge structure that facilitates perception and recall in the context of legal chess positions. However, when removing the underlying context of chess rules (i.e., positioning chess figures randomly on the board) recall performance in experts drops heavily (Gobet and Simon, 1996), which is plausible, as a central component that was constantly present during the acquisition of their automaticity was removed. Separating the encoding context from the performance context might tax execution.

In a computer simulation study, Botvinick and Plaut (2004) present a convincing rationale for how a degrading context can lead to action slips by modeling the naturalistic action of coffee making with a recurrent neural network (a type of neural network that can encode time and thus gain a form of memory). The model was trained to make coffee in a stable environment by chaining different subtasks together such as “add cream,” which then has subtasks such as fixating the necessary object, grabbing it, etc. Then, different levels of random variance were added to the (virtual) context, which led to the model producing more errors in proportion to the variance added—more variance, more errors. In a stable context (i.e., training context equals performance context), the model reliably produced coffee in a smooth sequence of correctly performed subtasks. Botvinick and Bylsma (2005) then tested their model with humans in an experimental setting with the exact same task. Participants were instructed to repeatedly make coffee in an 1-h laboratory session. Then, resembling variance (or destabilizing contexts), participants were interrupted randomly with subtraction tasks, which led, as predicted, to more subsequent action slips. Destabilizing contexts leads to a less fluent performance in routine tasks.

In the present study, we investigate the effect of context stability on the automatization of new habits and on the attainment of related habit goals. We refer to context stability here as the similarity of the current habit repetition context to the context in which the habit has routinely been performed in the past. We hypothesize that context stability leads to higher automaticity during habit performance. We expect these effects on the intraindividual (Level 1) as well as on the interindividual level (Level 2). A context stability factor that is applied to subjects should thus influence their average automaticity scores (Level-2-effect) and daily fluctuation in context stability should influence automaticity within subjects at that point in time (Level-1-effect).

*H1a*: Context stability increases automaticity on Level 1.

*H1b*: Context stability increases automaticity on Level 2.

So, it would be fair to assume that context stability should support behavioral automatization. Furthermore, we already have evidence that more automaticity leads to less motivational interference during habit performance (Stojanovic et al., 2020, 2021). This leads us to assume that context stability increases the degree to which the habit-repetition-specific goal is attained. In other words: When performing one’s habit as usual in the kitchen after lunch, it is more likely to reach a higher degree of the set goal for one habit repetition (habit repetition goal attainment; HRGA) than trying to perform one’s habit as an exception in a hotel room after dinner. As above, we expect effects on Level 1 and Level 2.

*H2a*: Context stability increases HRGA on Level 1.

*H2b*: Context stability increases HRGA on Level 2.

We further hypothesize that the effect of context stability on HRGA is mediated by automaticity.

*H3*: Automaticity mediates the effect of context stability on HRGA.

## The Present Study

H1a, H2a, and H3 are tested with two datasets, respectively, for better generalizability. Thus, the corresponding models will have a parameter for each dataset. The first dataset (Study 1) stems from a longitudinal diary study in which students built new study habits. In the first dataset, context stability was manipulated between subjects. With this context stability group factor, H1b and H2b are tested. The second dataset (Study 2) contains real user data from a habit building app (Stojanovic, 2019). Other variables of the second dataset have already been analyzed in another paper (Stojanovic et al., 2021), while the first dataset (Study 1) is analyzed for the first time in this paper. In both datasets, context stability, automaticity, and HRGA were measured as Level-1-variables after each habit repetition.

## Study 1: Habit Building With Manipulated Context Stability

### Materials and Methods

#### Participants

*N* = 95 university students (*M_age_* = 23.2, *SD_Age_* = 5.2; 82% female) participated in return for course credit. Participants with at least 12 habit repetitions were registered for an additional lottery for one of 10 online coupons worth 50 Euros each (~55 U.S. dollars). Participants who did not log at least one habit repetition were excluded from the data analysis. The participants were recruited in psychology lectures of two German universities and social media groups. 90% of the participants studied psychology and the remaining 10% something else.

#### Procedure and Measures

The data were collected with Qualtrics (Qualtrics, 2021).^1^ The study consisted of a pretest, a 6-week period with daily event sampling surveys and a posttest. In the pretest, participants were guided though the process of defining a new study habit in five (stable context condition), respectively, four (variable context condition) steps. First, participants defined a long-term goal they wanted to achieve over the 6-week period with the new study habit (e.g., “Worked through the whole textbook.” or “Having summarized all lectures of the statistics course”). Secondly, participants defined the anticipated duration of the habit, which was restricted to be between 3 and 30 min. Thirdly, participants in the stable context condition defined a context to perform the new habit in. Participants in the variable context condition skipped this step. The context consisted of a physical surrounding and a spot in the course of the day (e.g., “After brushing teeth in bed”). Fourthly, the learning activity of the habit was defined (e.g., “Work through textbook (read and mark).” or “Summarize lecture notes”). Finally, the habit repetition goal was defined, which indicates when a habit repetition can be considered completed (e.g., “Having worked through three pages of the textbook” or “Having summarized one lecture”). At the end of this habit definition process, participants were informed that the study investigated the influence of a stable/variable context during habit formation without conveying any information about expected effects.

Context stability was manipulated after the habit definition process by different instructions on where and when to perform the habit. Participants were instructed to do their habit at the same place (stable context) or to do their habit in different places such that at least the last five places would be different (variable context). Further, they were instructed to do their habit at the same time in the course of the day (stable context) or at different times in the course of the day such that at least the last five points in time in the course of the day would be different (variable context). Participants were reminded to keep their context stable/variable in this way in the daily habit repetition survey. All participants were instructed to perform their habit daily and answer the event sampling survey directly afterwards. Thereafter, participants answered items about constructs not relevant for this study.

After the pretest, the 6-week long event sampling phase started. The event sampling survey was accessible anytime via an individual link. Automaticity and context stability were measured on an 11-point scale (from *0 = does not apply at all* to *10 = applies perfectly*). HRGA was measured with one item (“How much percent of your daily habit goal did you achieve in your last habit repetition?”) and answered on a 0–100% scale. Automaticity was measured with the Self-Report Behavioral Automaticity Index (SRBAI; Gardner et al., 2012), a four-item automaticity subscale from the Self-Report Habit Index (SRHI; Verplanken and Orbell, 2003; e.g., “My habit is something I do automatically”). Context stability was measured with three items created by the authors, covering the physical place facet (“I did my last habit repetition in the same physical surroundings in which I usually did my earlier repetitions as well”), the time facet (“I did my last habit repetition at the same time in the course of the day at which I usually did my earlier repetitions as well”) and a general context item (“The context (physical as well as mental) of my last habit repetition was exactly the same as in the repetitions earlier”). Cronbach’s alpha for this 3-item context stability measure over all *N* = 2,482 habit repetitions not accounting for differences between persons was 0.90, with item-scale correlations of 0.78–0.81. The L1 reliability (within-person reliability) according to Nezlek (2017) and Bonito et al. (2012) was 0.65. L1 reliabilities are expected to have somewhat lower values than classical reliability measures and 0.65 can at least be classified as moderate (Nezlek, 2017). This within-person reliability indicates that any item’s context stability score on a given habit repetition measurement of a person can relatively accurately predict that person’s mean context stability score of that specific measurement point. The L2 reliability (between-person reliability) according to Nezlek (2017) and Bonito et al. (2012) was 0.98. This means, analogously, that any item’s average score of a person over all their habit repetitions can predict that person’s average context stability score over all their habit repetitions very precisely.

#### Data Analysis

The data structure is hierarchical with habit repetitions (Level 1) nested in persons (Level 2). We modeled growth curve models with multilevel regressions (Field, 2013) using IBM SPSS 27 to test H1a-b and H2a-b. Parameters were calculated with maximum likelihood estimation.

The growth curves (Model 1–6) describe the change of automaticity and HRGA over time (i.e., habit repetitions). Random intercepts (*u*_0_), random slopes (*u*_1_), and the covariance of the random intercepts and the random slopes (COV(*u*_0_,*u*_1_)) were specified with an unstructured covariance structure at the start of the estimation process.

We tested our mediation hypothesis H3 (context stability on automaticity on HRGA) by conducting a multilevel mediation analysis with the MLmed Beta 2 IBM SPSS macro by Rockwood (downloaded from https://njrockwood.com/mlmed; Rockwood, 2017). IBM SPSS 28 was used with MLmed. We specified random intercepts for the direct effects of context stability on automaticity and HRGA. Parameters were estimated with restricted maximum likelihood. In this analysis, the mediation is tested on both Level 1 and Level 2, so that each path will have a coefficient for each level of analysis. The MLmed macro centers Level-1-variables within-person to estimate within-effects. The between-effects of Level 2 are estimated by the mean values (e.g., the average HRGA value over all habit repetitions).

Model 1 (1) predicts automaticity at time t for person p with a random intercept *b*_0,*p*_, which is the average intercept of the sample *b*_00_ plus the individual deviation from that intercept *u*_0,*p*_ (2), plus the individual slope *b*_1,*p*_, which is the average slope for the effect of habit repetition (i.e., time) of the whole sample *b*_10_ plus the individual deviation from that slope *u*_1,*p*_ (3), times habit repetition plus habit repetition squared (habit repetition sq_t,p_) with a fixed beta (*b*_2_), which adds a quadratic trend, plus error_t,p_. We found in preliminary analyses that habit pausing (i.e., not performing the habit every day) had a negative influence on automaticity (both datasets) and potentially on HRGA (second dataset), which is why we also included habit pausing measured as days since the last habit repetition as a control variable in Model 1–6. For Model 2–6, *b*_0,*p*_ and *b*_1,*p*_ are the same as in Model 1.


(1) $$\begin{array}{l} Automaticity_{t,p}=b_{0,p}+b_{1,p}Habitrepetition_{t,p}\\ \;\;\;\;\;\;\;\;\;\;\;\;\;\;\;\;\;\;\;\;\;\;\;\;\;\;\;\;\;\;\;\;\;\;\;\;\;\;\;\;\;\;\;\;\;\;+b_{2}Habitrepetition\;sq_{t,p}\\ \;\;\;\;\;\;\;\;\;\;\;\;\;\;\;\;\;\;\;\;\;\;\;\;\;\;\;\;\;\;\;\;\;\;\;\;\;\;\;\;\;\;\;\;\;\;+b_{3}Habitpausing_{t,p}+\varepsilon_{t,p} \end{array}$$

(2) $$b_{0,p}=b_{00}+u_{0,p}$$

(3) $$b_{1,p}=b_{10}+u_{1,p}$$

In Model 2 (H1a), we added the Level 1 predictor context stability to Model 1, resulting in Eq. (4).


(4) $$\begin{array}{l} Automaticity_{t,p}=b_{0,p}+b_{1,p}Habitrepetition_{t,p}\\ \;\;\;\;\;\;\;\;\;\;\;\;\;\;\;\;\;\;\;\;\;\;\;\;\;\;\;\;\;\;\;\;\;\;\;\;\;\;\;\;\;\;\;\;\;\;+b_{2}Habitrepetition\;sq_{t,p}\\ \;\;\;\;\;\;\;\;\;\;\;\;\;\;\;\;\;\;\;\;\;\;\;\;\;\;\;\;\;\;\;\;\;\;\;\;\;\;\;\;\;\;\;\;\;\;+b_{3}Habitpausing_{t,p}\\ \;\;\;\;\;\;\;\;\;\;\;\;\;\;\;\;\;\;\;\;\;\;\;\;\;\;\;\;\;\;\;\;\;\;\;\;\;\;\;\;\;\;\;\;\;\;+b_{5}Contextstability_{t,p}+\varepsilon_{t,p} \end{array}$$

In Model 3 (H1b), we added the dummy coded group variable context stability group (0 = stable context condition, 1 = variable context condition) as a Level 2 predictor to Model 1, resulting in Eq. (5).


(5) $$\begin{array}{l} Automaticity_{t,p}=b_{0,p}+b_{1,p}Habitrepetition_{t,p}\\ \;\;\;\;\;\;\;\;\;\;\;\;\;\;\;\;\;\;\;\;\;\;\;\;\;\;\;\;\;\;\;\;\;\;\;\;\;\;\;\;\;\;\;\;\;\;+b_{2}Habitrepetition\;sq_{t,p}\\ \;\;\;\;\;\;\;\;\;\;\;\;\;\;\;\;\;\;\;\;\;\;\;\;\;\;\;\;\;\;\;\;\;\;\;\;\;\;\;\;\;\;\;\;\;\;+b_{3}Habitpausing_{t,p}\\ \;\;\;\;\;\;\;\;\;\;\;\;\;\;\;\;\;\;\;\;\;\;\;\;\;\;\;\;\;\;\;\;\;\;\;\;\;\;\;\;\;\;\;\;\;\;+b_{6}Contextstabilitygroup_{p}+\varepsilon_{t,p} \end{array}$$

Model 4 predicts HRGA at time *t* for person *p* with habit repetition (i.e., time), habit pausing, and automaticity plus error_t,p_.


(6) $$\begin{array}{l} HRGA_{t,p}=b_{0,p}+b_{1,p}Habitrepetition_{t,p}\\ \;\;\;\;\;\;\;\;\;\;\;\;\;\;\;\;\;\;\;\;\;\;\;\;\;\;\;\;\;+b_{3}Habitpausing_{t,p}\\ \;\;\;\;\;\;\;\;\;\;\;\;\;\;\;\;\;\;\;\;\;\;\;\;\;\;\;\;\;+b_{4}Automaticity_{t,p}+\varepsilon_{t,p} \end{array}$$

In Model 5 (H2a), we added the Level 1 predictor context stability to Model 4, resulting in Eq. (7).


(7) $$\begin{array}{l} HRGA_{t,p}=b_{0,p}+b_{1,p}Habitrepetition_{t,p}\\ \;\;\;\;\;\;\;\;\;\;\;\;\;\;\;\;\;\;\;\;\;\;\;\;\;\;\;\;+b_{3}Habitpausing_{t,p}+b_{4}Automaticity_{t,p}\\ \;\;\;\;\;\;\;\;\;\;\;\;\;\;\;\;\;\;\;\;\;\;\;\;\;\;\;\;+b_{5}Contextstability_{t,p}+\varepsilon_{t,p} \end{array}$$

In Model 6 (H2b), we added the group variable context stability group as a Level 2 predictor to Model 4, resulting in Eq. (8).


(8) $$\begin{array}{l} HRGA_{t,p}=b_{0,p}+b_{1,p}Habitrepetition_{t,p}\\ \;\;\;\;\;\;\;\;\;\;\;\;\;\;\;\;\;\;\;\;\;\;\;\;\;\;\;\;+b_{3}Habitpausing_{t,p}+b_{4}Automaticity_{t,p}\\ \;\;\;\;\;\;\;\;\;\;\;\;\;\;\;\;\;\;\;\;\;\;\;\;\;\;\;\;+b_{6}Contextstabilitygroup_{p}+\varepsilon_{t,p} \end{array}$$

### Results

#### Preliminary Findings

The dataset contained *N* = 2,482 habit repetitions of *N* = 95 participants with an average of *M* = 26.13 (*SD* = 12.20) logged habit repetitions per participant. Concerning pauses between habit repetitions (i.e., not doing a habit repetition for at least 1 day), with *n* = 1,780 (74.6%) the majority of habit repetitions were done without pause. There was no association of average context stability and average habit pausing in either group, *r*_stable_ = −0.11, *p* = 0.49, *r*_variable_ = −0.20, *p* = 0.18. See Table 1 for a comparison of the descriptive statistics for each group.

**Table 1:** Descriptive statistics for the stable and variable context group.

	Variable context group	Stable context group	Both groups
Participants (L2)	*n* = 49	*n* = 46	*N* = 95
Total habit repetitions (L1)	*n* = 1,207	*n* = 1,275	*N* = 2,482
**Habit pausing**
no pause	*n* = 838 (72.4%)	*n* = 942 (76.6%)	*n* = 1,780 (74.6%)
1 day	*n* = 190 (16.4%)	*n* = 200 (16.3%)	*n* = 390 (16.3%)
2 days	*n* = 63 (5.4%)	*n* = 44 (3.6%)	*n* = 107 (4.5%)
>2 days	*n* = 67 (5.8%)	*n* = 43 (3.5%)	*n* = 110 (4.6%)
***M* (*SD*) and *Mdn***
Habit repetitions	24.63 (12.06)	27.72 (12.27)	26.13 (12.20)
	25.00	34.00	29.00
Context stability	2.99 (1.34)	8.13 (1.32)	5.47 (2.90)
	3.00	9.00	6.00
Automaticity	3.75 (1.90)	4.41 (2.34)	4.07 (2.14)
	3.75	4.50	4.00
HRGA	72.69 (21.70)	74.84 (22.31)	73.73 (21.91)
	92.00	100.00	95.00

In the lower half of the table, the means and standard deviations are in the upper lines and medians are in the lower lines, respectively. For habit repetitions, the accumulated total habit repetition count was relevant, so the means and medians were calculated by averaging the total habit repetition count over persons. The remaining means were calculated by averaging the mean of each person (the mean of means). The remaining medians were calculated over all habit repetitions. L2 = Level 2, person-level; L1 = Level 1, habit repetition level; HRGA = habit repetition goal attainment.

#### Manipulation Check

Participants in the stable context group reported on average a significantly higher context stability than participants of the variable context group, *d* = 1.33, *t*(−18.80) = 4.21, *p* < 0.001 (see Table 1). Hence, the context stability manipulation was successful.

#### Automaticity and Context Stability

H1a-b aim at testing context stability as a predictor for automaticity in the habit formation process. First, we describe and test the automaticity baseline model representing habit formation over time. Thereafter, we add context stability as a Level 1 predictor to test H1a and then the Level 2 context stability group predictor to test H1b.

##### Automatization Over Time

To model habit formation over time, automaticity was predicted with habit repetition as the time variable with random slopes and random intercepts. Then, habit repetition was squared and added as a predictor to model decreasing automaticity gains in higher habit repetition ranges. Finally, the time since the last habit repetition in days, habit pausing, was added as a control variable (see Table 2, Model 1, upper values). This pattern replicates a typical habit growth trajectory with steep automaticity gains at the beginning of the habit building process and asymptotically decreasing automaticity growth in the higher repetition range (Lally et al., 2010; Stojanovic et al., 2020, 2021) and constitutes the automaticity baseline model (Model 1). However, this typical habit growth trajectory could not be replicated with this dataset, as habit repetition squared had no influence on automaticity, *b* = −0.0004 (*SE* = 0.0003), *t*(2285.45) = −1.26, *p* = 0.209. Neither age, *b* = −0.068 (*SE* = 0.039), *t*(92.57) = −1.76, *p* = 0.082, nor gender, *b* = 0.593 (*SE* = 0.514), *t*(92.54) = 1.15, *p* = 0.251, had an influence on automaticity.

**Table 2:** Multilevel regressions of automaticity on context stability based on controlled study data with manipulated context stability (Study 1, upper value) and real-life user data (Study 2, lower value).

Parameter	Model 1			Model 2 (H1a)			Model 3 (H1b)				Automaticity	
	Estimate	*SE*	95% CI	Estimate	*SE*	95% CI	Estimate	*SE*	95% CI
**Fixed effects**
Intercept (*b*_00_)	2.937^***^	0.215	2.511, 3.363	2.550^***^	0.224	2.106, 2.994	3.149^***^	0.299	2.556, 3.742
	3.676^***^	0.136	3.499, 4.034	2.511^***^	0.236	2.047, 2.975	—	—	—
**Level 1**									
Habit repetition (*b*_10_)	0.098^***^	0.014	0.070, 0.125	0.096^***^	0.014	0.068, 0.123	0.098^***^	0.014	0.070, 0.125
	0.164^***^	0.009	0.147, 0.181	0.146^***^	0.010	0.127, 0.165	—	—	—
Habit repetition sq. (*b*_2_)	−0.0004	0.0003	−0.0009, 0.0002	−0.0003	0.0003	−0.0009, 0.0002	−0.0004	0.0003	−0.0009, 0.0002
	−0.0014^***^	0.0001	−0.0017, −0.0012	−0.0013^***^	0.0001	−0.0015, −0.0010	—	—	—
Habit pausing (*b_3_*)	−0.098^***^	0.018	−0.134, −0.062	−0.094^***^	0.018	−0.130, −0.058	−0.098^***^	0.018	−0.134, −0.062
	−0.008^**^	0.003	−0.013, −0.002	−0.007^*^	0.003	−0.013, −0.002	—	—	—
Context stability (*b_5_*)				0.073^***^	0.013	0.048, 0.099			
				0.020^***^	0.002	0.015, 0.024			
**Level 2**									
Context stability group (*b_6_*)							−0.411	0.405	−1.215, 0.394
							—	—	—
**Random effects**
Random intercept (VAR *u*_0_)	3.756^***^	0.598	2.750, 5.131	3.710^***^	0.590	2.717, 5.066	3.712^***^	0.591	2.717, 5.072
	2.480^***^	0.317	1.930, 3.186	3.131^***^	0.468	2.336, 4.197	—	—	—
Cov. rand. intercept, rand. slope (COV *u*_0_, *u*_1_)	−0.018	0.022	−0.061, 0.025	−0.020	0.022	−0.062, 0.022	−0.017	0.022	−0.060, 0.026
	—	—	—	—	—	—	—	—	—
Random slope (VAR *u*_1_)	0.007^***^	0.001	0.005, 0.010	0.007^***^	0.001	0.005, 0.010	0.007^***^	0.001	0.005, 0.010
	—	—	—	—	—	—	—	—	—

For each line, the upper value refers to Study 1 and the lower to Study 2. All estimated coefficients are unstandardized. Variables as specified in the model equations are in parentheses. The coding for Context stability group is 0 = stable context and 1 = variable context. CI = confidence interval; Habit repetition sq = habit repetition squared; VAR = variance; COV = covariance; Cov. rand. intercept, rand. slope = covariation of the random intercept and the random slope.

* *p* < 0.05;

** *p* < 0.01;

*** *p* < 0.001.

##### Automatization and Context Stability on Level 1

To test H1a, the influence of Level 1 context stability on automaticity, context stability was added to the automaticity baseline model (Model 1) as a predictor, resulting in Model 2. As expected, context stability predicted automaticity, *b* = 0.073 (*SE* = 0.013), *t*(2340.98) = 5.62, *p* < 0.001.

##### Automatization and Manipulated Context Stability

To test H1b, the influence of the context stability manipulation on automaticity, context stability group was added to the automaticity baseline model (Model 1) as a dummy coded Level 2 predictor, resulting in Model 3. Contrary to our expectation, the group factor did not predict automaticity, *b* = −0.411 (*SE* = 0.405), *t*(93.62) = −1.01, *p* = 0.313.

#### HRGA and Context Stability

H2a-b aim at testing context stability as a predictor for HRGA in the habit formation process. First, we defined a HRGA baseline model with habit repetition, habit pausing, and automaticity (see Table 3, Model 4, upper values). Automaticity positively predicted HRGA, *b* = 4.360 (*SE* = 0.346), *t*(1277.37) = 12.59, *p* < 0.001. Habit repetition, *b* = 0.058 (*SE* = 0.099), *t*(53.27) = 0.59, *p* = 0.561, and habit pausing, *b* = −0.370 (*SE* = 0.334), *t*(2349.01) = −1.11, *p* = 0.268, did not predict HRGA. Then, to test H2a, we added context stability (see Table 3, Model 5, upper values) as a Level 1 predictor. As expected, context stability positively predicted HRGA, *b* = 1.816 (*SE* = 0.232), *t*(2180.30) = 7.82, *p* < 0.001. Finally, to test H2b, we added the group factor context stability group as a Level 2 predictor to the HRGA baseline model (Model 4), resulting in Model 6. Contrary to our expectation, the group factor did not predict HRGA, *b* = 0.076 (*SE* = 4.333), *t*(87.22) = 0.02, *p* = 0.986.

**Table 3:** Multilevel regressions of HRGA on context stability based on controlled study data with manipulated context stability (Study 1, upper value) and real-life user data (Study 2, lower value).

Parameter	Model 4			Model 5 (H2a)			Model 6 (H1b)				HRGA	
	Estimate	*SE*	95% CI	Estimate	*SE*	95% CI	Estimate	*SE*	95% CI
**Fixed effects**
Intercept (*b*_00_)	56.120^***^	2.972	50.235, 62.005	47.340^***^	3.194	41.029, 53.652	56.080^***^	3.753	48.656, 63.505
	62.179^***^	2.341	57.559, 66.779	54.756^***^	3.009	48.823, 60.868	—	—	—
**Level 1**									
Habit repetition (*b*_10_)	0.058	0.099	−0.140, 0.256	0.044	0.098	−0.152, 0.240	0.058	0.099	−0.140, 0.256
	1.330^***^	0.196	0.933, 1.728	0.877^***^	0.205	0.468, 1.282	—	—	—
Habit pausing (*b_3_*)	−0.370	0.334	−1.026, 0.286	−0.278	0.331	−0.926, 0.371	−0.370	0.334	−1.026, 0.286
	−0.070^*^	0.028	−0.124, −0.016	−0.064^*^	0.026	−0.116, −0.014	—	—	—
Automaticity (*b_4_*)	4.360^***^	0.346	3.680, 5.039	4.059^***^	0.346	3.381, 4.738	4.360^***^	0.346	3.680, 5.039
	1.820^***^	0.209	1.411, 2.229	1.335^***^	0.200	0.943, 1.727	—	—	—
Context stability (*b_5_*)				1.816^***^	0.232	1.360, 2.271			
				0.210^***^	0.020	0.172, 0.249	—	—	—
**Level 2**									
Context stability group (*b_6_*)							0.076	4.333	−8.537, 8.689
**Random effects**
Random intercept (VAR *u*_0_)	631.74^***^	106.46	454.04, 878.99	648.84^***^	109.00	466.81, 901.86	631.73^***^	106.46	454.04, 878.98
	831.26^***^	102.54	652.74, 1,058.60	805.75^***^	120.57	600.94, 1,080.36	—	—	—
Cov. rand. intercept, rand. slope (COV *u*_0_, *u*_1_)	−10.92^***^	3.070	−16.938, −4.904	−10.520^**^	3.087	−16.570, −4.470	−10.92^***^	3.070	−16.937, −4.902
	−46.61^***^	8.370	−63.01, −30.20	−46.723^***^	8.868	−64.104, −29.342	—	—	—
Random slope (VAR *u*_1_)	0.471^**^	0.141	0.262, 0.848	0.458^**^	0.139	0.253, 0.832	0.471^***^	0.141	0.262, 848
	3.002^***^	0.700	1.900, 4.741	2.924^***^	0.674	1.860, 4.594	—	—	—

For each line, the upper value refers to Study 1 and the lower to Study 2. All estimated coefficients are unstandardized. Variables as specified in the model equations are in parentheses. The coding for Context stability group is 0 = stable context and 1 = variable context. HRGA = habit repetition goal attainment; CI = confidence interval; VAR = variance; COV = covariance; Cov. rand. intercept, rand. slope = covariation of the random intercept and the random slope.

* *p* < 0.05;

** *p* < 0.01;

*** *p* < 0.001.

#### Automaticity Mediates the Effect of Context Stability on HRGA

**Figure 1:**
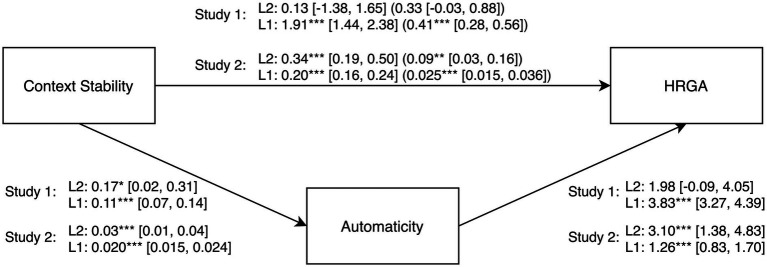
Multilevel mediation analysis with unstandardized regression coefficients of the effect of context stability on HRGA (habit repetition goal attainment) through automaticity. The first coefficients on the path from context stability to HRGA represent the direct effect without the mediator; the coefficients in parentheses on this path represent the indirect effects with the mediator included in the model. The random intercepts were significant for both automaticity (Study 1: variance *u*_0automaticity_ = 4.21*** [3.12, 5.68]; Study 2: variance *u*0_automaticity_ = 3.41*** [2.68, 4.34]), and HRGA (Study 1: variance *u*0_HRGA_ = 407.69*** [296.76, 560.08]; Study 2: variance *u*0_HRGA_ = 491.90*** [389.32, 621.52]). Level 2 (L2) = person-level (Study 1)/habit-level (Study 2); Level 1 (L1) = habit repetition level. 95% confidence intervals are in brackets. *p < 0.05, **p < 0.01, ***p < 0.001.

H3 aims at testing automaticity as a mediator of the effect of context stability on HRGA. We conducted a multilevel mediation analysis with the MLmed Beta 2 macro by Rockwood (downloaded from https://njrockwood.com/mlmed; Rockwood, 2017). We specified random intercepts for the direct effects of context stability on automaticity and HRGA. As expected, automaticity mediated the influence of context stability on HRGA. We found this effect on Level 1 (within-indirect effect), *b* = 0.412 (*SE* = 0.072, *CI* = 0.276, 0.557), *p* < 0.001. We found no such effect on Level 2 (between-indirect effect) with averaged Level 1 data, *b* = 0.330 (*SE* = 0.240, *CI* = −0.026, 0.876), *p* = 0.170. The CIs for the indirect effects were estimated using Monte Carlo simulations with 10.000 samples. See Figure 1 for the complete mediation analysis.

#### Auxiliary Analyses

To investigate the unexpected disparity between context stability affecting automaticity and HRGA on Level 1 but not on Level 2, we fitted Model 2 (automaticity) and Model 5 (HRGA) separately to the stable and the variable context group. The results are summarized in Table 4. In Model 2, context stability on Level 1 predicts automaticity in the stable context group, *b* = 0.178 (*SE* = 0.018), *t*(1175.73) = 10.03, *p* < 0.001, but not in the variable context group, *b* = −0.012 (*SE* = 0.019), *t*(1103.68) = −0.64, *p* = 0.524. In the stable context group, habit repetition squared predicts automaticity, *b* = −0.0011 (*SE* = 0.0003), *t*(1184.10) = −3.48, *p* < 0.001, which it did not in the variable context group, *b* = 0.0005 (*SE* = 0.0004), *t*(1101.51) = 1.06, *p* = 0.290. In the main analysis above with pooled groups, habit repetition squared was not significant (see Table 2, Model 1–3, upper values), seemingly not replicating the expected asymptotic habit growth curve. In Model 5, context stability on Level 1 predicts HRGA in the stable context group, *b* = 4.902 (*SE* = 0.322), *t*(1216.27) = 15.25, *p* < 0.001, but not in the variable context group, *b* = −0.351 (*SE* = 0.348), *t*(1141.80) = −1.01, *p* = 0.312.

**Table 4:** Auxiliary multilevel regressions of automaticity and HRGA on context stability for each group based on controlled study data with manipulated context stability (Study 1).

Parameter	Model 2			Model 5		
	Automaticity			HRGA		
	Estimate	*SE*	95% CI	Estimate	*SE*	95% CI
**Fixed effects**	Intercept (*b*_00_)
	1.561^***^	0.345	0.872, 2.250	21.706^***^	4.841	12.078, 31.333
	2.930^***^	0.296	2.339, 3.522	58.146^***^	4.078	50.019, 66.274
**Level 1**						
Habit repetition (*b*_10_)	0.120^***^	0.018	0.084, 0.155	0.071	0.127	−0.190, 0.333
	0.075^***^	0.021	0.033, 0.116	0.099	0.138	−0.185, 0.383
Habit repetition sq. (*b*_2_)	−0.0012^***^	0.0003	−0.0019, −0.0005			
	0.0005	0.0004	−0.0004, 0.0013			
Habit pausing (*b_3_*)	−0.064^*^	0.025	−0.113, −0.015	−0.255	0.435	−1.107, 0.598
	−0.117^***^	0.026	−0.168, −0.066	−0.336	0.471	−1.261, 0.588
Automaticity (*b_4_*)				2.921^***^	0.449	2.038, 3.804
				3.997^***^	0.498	3.020, 4.975
Context stability (*b_5_*)	0.178^***^	0.018	0.143, 0.213	4.902^***^	0.322	4.271, 5.533
	−0.012	0.019	−0.050, 0.025	−0.351	0.348	−1.033, 0.331
**Random effects**
Random intercept (VAR *u*_0_)	4.037^***^	0.916	2.588, 6.296	696.34^***^	163.06	440.05, 1101.91
	3.456^***^	0.764	2.240, 5.331	543.70^***^	130.75	339.35, 871.09
Cov. rand. intercept, rand. slope (COV *u*_0_, *u*_1_)	−0.009	0.033	−0.073, 0.056	−13.683^**^	4.663	−22.821, −4.544
	−0.033	0.030	−0.092, 0.025	−8.885^*^	3.702	−16.141, −1.628
Random slope (VAR *u*_1_)	0.006^***^	0.002	0.004, 0.010	0.455^*^	0.179	0.210, 0.984
	0.008^***^	0.002	0.005, 0.015	0.401^*^	0.181	0.165, 0.970

For each line, the upper value refers to the stable context group and the lower to the variable context group. All estimated coefficients are unstandardized. Variables as specified in the model equations are in parentheses. HRGA = habit repetition goal attainment; CI = confidence interval; VAR = variance; COV = covariance; Cov. rand. intercept, rand. slope = covariation of the random intercept and the random slope.

* *p* < 0.05;

** *p* < 0.01;

*** *p* < 0.001.

**Figure 2:**
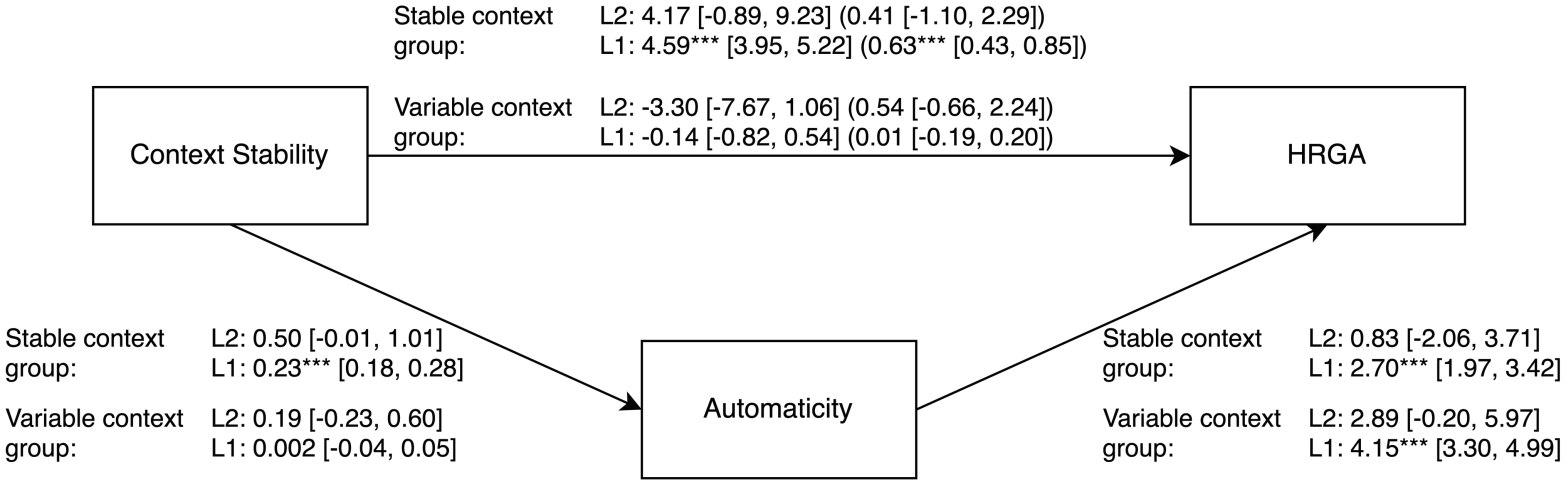
Multilevel mediation analysis of the effect of context stability on HRGA (habit repetition goal attainment) through automaticity with Study 1 dataset comparing stable and variable context groups with unstandardized regression coefficients. The first coefficients on the path from context stability to HRGA represent the direct effect without the mediator; the coefficients in parentheses on this path represent the indirect effects with the mediator included in the model. The random intercepts were significant for both automaticity (Stable context group: variance *u*_0automaticity_ = 4.99*** [3.24, 7.69]; Variable context group: variance *u*_0automaticity_ = 3.46*** [2.26, 5.28]), and HRGA (Stable context group: variance *u*_0HRGA_ = 436.51*** [277.34, 687.02]; Variable context group: variance *u*_0HRGA_ = 362.25*** [229.01, 573.00]). Level 2 (L2) = person-level; Level 1 (L1) = habit repetition level. 95% confidence intervals are in brackets. ***p < 0.001.

Further, we also repeated the multilevel mediation analysis for the stable and variable context group separately with the same specifications as in the main analysis. On Level 1 (within-indirect effect), automaticity mediated the influence of context stability on HRGA in the stable context group, *b* = 0.626 (*SE* = 0.108, *CI* = 0.427, 0.846), *p* < 0.001, but not in the variable context group, *b* = 0.009 (*SE* = 0.010, *CI* = −0.186, 0.203), *p* = 0.926. On Level 2 (between-indirect effect) with averaged Level 1 data, there was neither an effect in the stable context group, *b* = 0.414 (*SE* = 0.829, *CI* = −1.100, 2.294), *p* = 0.617, nor in the variable context group, *b* = 0.543 (*SE* = 0.730, *CI* = −0.659, 2.245), *p* = 0.457. See Figure 2 for the complete mediation analyses.

### Short Discussion

As to our knowledge, this study is the first in which context stability was actively manipulated to then track its influence on the development of new habits. As expected, we found that habit building worked better in stable contexts as indicated by higher automaticity scores. However, as the auxiliary analyses show, context stability only seems to predict automaticity on higher levels past a certain threshold: In the stable context group, higher context stability on Level 1 predicted higher automaticity, but it had no effect in the variable context group. This effect, rooted in the stable context group, was strong enough to spill over into the pooled analysis with both groups, which explains why context stability on Level 1 could become a significant predictor for automaticity in the main analysis, while the Level 2 context stability group factor—which resembles a considerable difference of average Level 1 context stability—would have no such effect. In generally stable contexts, it matters how stable it is precisely in a given day and small deviations will tax automaticity. In generally variable contexts with new times in the flow of the day and new places, there is no association with the behavior and thus there seems to be no association of automaticity and context.

On the other hand, it needs to be noted that even with the imposed friction of varying contexts, participants of the variable context group were able to build considerable degrees of automaticity by repetition. One needs to take into account however, that participants knew they were in a controlled study with instructions to perform their defined habits in certain ways and had course credit on the line for participation. We could not control if and how participants reminded themselves to do their habits. Gardner et al. (2016) show that instigation automaticity predicts actual frequency of behavior, but execution automaticity does less so. Outside of a study setting, very unstable contexts might have a higher risk of failing to instigate habit execution in the first place even if the following habit execution pattern might be moderately automized, which would mean that the habit repetition count in the variable context group might be overestimated.

Another source of potential systematic error variance is the instruction for participants of the variable context group to deliberately switch contexts before their study performance. This instruction triggers planning of the study behavior that lies before them as conscious decisions on the time and place for each repetition need to be made before each execution, which could have created an intentional focus on the task at hand, potentially resulting in increased situational intention and deliberation concerning the target behavior. As Ouellette and Wood (1998) famously showed, intention is a stronger predictor for future behavior if the behavior was performed seldomly and in varying contexts in the past, which is, next to the frame of participating in an empirical study, a second possible factor that might have increased the habit repetition count in the variable context group. Thus, a milder manipulation of context stability, which would not trigger planning, intention, and deliberation, might result in *less* total habit repetitions. This potentially confounding deliberation factor should be considered when interpreting the reported results which include data from the variable context group.

Furthermore, we found that when habit repetitions were performed in stable contexts, HRGA was higher. Put differently, habits—respectively the behaviors participants set out to make a habit at the beginning of the study—were quit more often before completion when performed in varying contexts. The auxiliary analyses provide a more precise picture again. Analogously to automaticity, context stability on Level 1 predicted HRGA in the stable context group, but not in the variable context group, again resulting in a weaker spillover effect in the main analysis with pooled data. We found this effect to be partially mediated by automaticity. Again, the auxiliary analyses revealed that this mediation can only be found in the stable context group. However, the automaticity-HRGA path is significant for both groups. Irrespective of how automaticity is obtained, it seems to increase HRGA. Automaticity is associated with less want conflicts (i.e., wanting to do something else) and less motivational interference (Stojanovic et al., 2020) during habit performance and with more habit-specific self-efficacy (Stojanovic et al., 2021). Both want conflicts and motivational interference are negatively related to self-regulation (Fries et al., 2008; Grund and Fries, 2014), while self-efficacy is strongly associated with self-regulation (Luszczynska et al., 2005). When considering this, the path of stable contexts leading to more automaticity (in the stable context group), which leads to a higher degree of HRGA, is plausible.

With study 2, we want to stress test our hypotheses with real user data of a habit building app that was developed and published as a product of earlier research (Stojanovic et al., 2020).

## Study 2: User Data Insights on Context Stability During Habit Building

### Materials and Methods

#### Participants

Here, we analyzed user data from an app we published in the AppStore (“Grow - Habit Builder”; Stojanovic, 2019) after validation with a closed beta version in earlier research (Stojanovic et al., 2020). The closed beta dataset (Stojanovic et al., 2020) and this user dataset of Study 2 are two separate datasets with no overlap. The dataset contained *N* = 2,368 habit repetitions (Level 1) of *N* = 218 users, who defined and tracked a total of *N* = 308 habits (Level 2) from 17.09.18–04.02.21. Habits with less than two habit repetitions as well as habits from the first author had previously been removed from the dataset. The data stems mainly from participants from Germany (25.3%), India (21.4%) and the United States(13.4%). The rest of the data was logged from countries all over the world with each contributing less than 4% of the total. 48.6% of users were female. 50% of the users were in the age group of 18–24 years, 31.8% were in the age group of 25–34 years, 12.8% were in the age group of 35–44 years, and 5.4% were in the age group of 45–54 years.^2^

#### Procedure and Measures

The habit definition process of the AppStore app “Grow - Habit Builder” was similar to the one of Study 1 in the stable context condition with some additions and usability modifications. In a first step, the users gave their habit a (1) *name*. Then, as in Study 1, users were asked to define the (2) *long-term goal* they want to achieve with the habit. When defining the estimated (3) *duration*, users were not constrained to a certain time as in Study 1, but the app recommended a range of 3–60 min. Users were informed that they should stop their habit only after having reached their habit action goal (Step 6) and not after the defined duration. Defining a duration was supposed to help the users planning their habit and integrating it more easily in the flow of their day. Excluding 20 outlier habits with over 120 min as a duration goal, the average defined duration was *M_duration_* = 23.72 (*SD_duration_* = 23.39) with a median of *Mdn_duration_* = 15. Then, users defined the (4) *context* for their habit by specifying a time in the course of the day (e.g., “after brushing teeth”) and a physical environment (e.g., “lying in bed”). Then, users defined the (5) *habit action* (analogous to the learning activity in Study 1) by specifying the action they would perform during the habit (e.g., “Write blog content” or “Solve coding problem on leetcode [a programming learning website]”). Users were not restricted to study habits as in Study 1. Next, users defined a (6) *habit action goal* (analogous to the habit repetition goal in Study 1), which would render a current habit repetition completed after achieving (e.g., “Write at least 200 words” or “Solve one coding problem and determine best practice”). After that, users were asked to define a short version of their habit, which they should perform instead of the normal habit if they were under time pressure or feel they would not be able to perform their full habit for any other reason. The so-called (7) *emergency habit action* and the (8) *emergency habit action goal* were defined analogously to steps 5 and 6 but with the instruction to make it considerably shorter and be able to perform it anywhere if possible. Two hundred forty-eight (10.5%) of the 2,368 analyzed habit repetitions were marked as emergency habit repetitions. As emergency habit repetitions were used sparingly and are similar to the normal version of the habit, they were treated as normal habit repetitions in data analysis. In the (9) *frequency*-step, users chose the weekdays on which they wanted to perform the habit and were thus not instructed to perform their habit daily as in Study 1. However, the app recommended setting the goal of performing the habit daily, which was done in 258 (83.8%) of the 308 analyzed habits. As a second part of this step, the users chose a time on which they wanted to be reminded of their new habit on the chosen weekdays. Finally, the app showed the users a summary of the habit and adaptions could be made for each step before saving the new habit.

Having finished the habit definition process, users could log data for their habit after each habit repetition. The event sampling process was the same as in Study 1, however with an adaption for efficiency and usability. The app tracks several scales, which are not all relevant to this article. In order to keep break-off rates low, which rise with questionnaire length in mobile surveys (Mavletova and Couper, 2015), we adapted the event sampling procedure. Instead of presenting all items from all scales in every measurement, random items from each scale were presented. With a total of 9–13 items per measurement and items from all scales, the users could quickly log their data providing information for all tracked scales. Modularization of longer questionnaires in shorter ones in mobile surveys does not reduce data quality and can even lead to less missing data and reduce satisficing (Toepoel and Lugtig, 2018). Among other constructs not relevant to this investigation, automaticity, context stability, and HRGA were measured. This measurement adaption only applies to automaticity, which was measured on a 11-point scale (from *0 = strongly disagree* to *10 = strongly agree*) with 10 partially adapted items from the SRHI^3^ (Verplanken and Orbell, 2003; e.g. “This habit is something I do automatically”). In the first five repetitions, automaticity was measured with 3–4 items and with two items in all following repetitions. Context stability [“Compared to your earlier habit repetitions: How similar was the context of THIS habit repetition (environment, time of day, people around you etc.) to your usual habit context from earlier repetitions?”] and HRGA [“How much of your habit action goal (the goal you set for ONE REPETITION) did you attain today?”] were measured after habit repetitions with one item, respectively, after every habit repetition and answered on a 0–100% scale. HRGA was measured after every habit repetition, while context stability was measured from the third habit repetition onward.

#### Data Analysis

We fitted the same models to this dataset that we also fitted to the dataset of Study 1 apart from Model 3 (H1b) and 6 (H2b), as they contain the group variables for context stability which do not exist in the dataset of Study 2. Here, habit repetitions (Level 1) are nested in habits (Level 2), which are nested in persons (Level 3). However, most users in the analyzed dataset only logged data for one habit (*n* = 166; 76%). We modeled the user data with Level 1 and Level 2 as in Study 1 and then performed exactly the same calculations.

### Results

#### Preliminary Findings

The dataset contained *N* = 2,368 habit repetitions of *N* = 308 defined habits, with an average of *M* = 13.90 (*SD* = 18.16) logged habit repetitions per habit. Most of the habits were related to studying (e.g., working through study material after the evening snack; learning Spanish with a language learning app before going to bed), physical exercise (e.g., pushups before going to work; video-guided yoga before breakfast) and mental focusing practices (e.g., meditation after lunch; breathing exercises while having a cold shower in the morning). Concerning habit pausing, with *n* = 1,328 (64.5%) the majority of habit repetitions were done without pause, *n* = 315 (15.3%) were done with a pause of 1 day, *n* = 137 (6.7%) were done with a pause of 2 days, and *n* = 280 (13.6%) were done with a pause of 3 days or more. Over all *N* = 2,368 habit repetitions, mean HRGA was *M* = 83.41% (*SD* = 28.20) with a median of *Mdn* = 100%. Over *n* = 1,720 habit repetitions (context stability was not measured after the first three repetitions), context stability was *M* = 80.88% (*SD* = 25.18) with a median of *Mdn* = 92%. There was no association of average context stability and average habit pausing, *r* = 0.08, *p* = 0.26.

#### Automaticity and Context Stability

Retesting H1a, we performed the same analyses as in Study 1. First, we tested the automaticity baseline model representing habit formation over time with this second dataset. Then, we added context stability as a predictor to retest H1a.

##### Automatization Over Time

We could replicate the automaticity baseline model from Study 1 with similar results, but this time with the expected significant coefficient for habit repetition squared, *b* = −0.0014 (*SE* = 0.0001), *t*(1983.24) = −13.46, *p* < 0.001. Furthermore, we found that the random slope was redundant, which is why we removed it from the model (see Table 2, Model 1, lower value).

##### Automatization and Context Stability

To retest H1a, the influence of context stability on automaticity, context stability was added to the automaticity baseline model (Model 1) as a Level 1 predictor, resulting in Model 2. As expected, context stability predicted automaticity, *b* = 0.020 (*SE* = 0.002), *t*(1714.00) = 8.88, *p* < 0.001.

#### HRGA and Context Stability

Retesting H2a, we performed the same analyses as in Study 1. First, we tested the HRGA baseline model with habit repetition and automaticity as predictors with this second dataset (see Table 3, Model 4, lower values). Automaticity positively predicted HRGA, *b* = 1.820 (*SE* = 0.209), *t*(1634.65) = 8.73, *p* < 0.001. Then, we added context stability (see Table 3, Model 5, lower values) as a predictor to retest H2a. As expected, context stability positively predicted HRGA, *b* = 0.210 (*SE* = 0.020), *t*(1672.42) = 10.70, *p* < 0.001.

#### Automaticity Mediates the Effect of Context Stability on HRGA

Retesting H3, we performed the same analyses as in Study 1. As expected, automaticity mediated the influence of context stability on HRGA. We found this effect on Level 1 (within-indirect effect), *b* = 0.025 (*SE* = 0.005, *CI* = 0.015, 0.036), *p* < 0.001, as well as on Level 2 (between-indirect effect) with averaged Level 1 data, *b* = 0.087 (*SE* = 0.033, *CI* = 0.032, 0.157), *p* < 0.01. The CIs for the indirect effects were estimated using Monte Carlo simulations with 10,000 samples. See Figure 1 for the complete mediation analysis.

### Short Discussion

In Study 2, we found corroborating empirical evidence for H1a, H2a and H3. As expected, stable habit contexts predicted higher automaticity and higher HRGA. Further, we could replicate the mediation of the effect of context stability on HRGA via automaticity. In Study 2 however, we also found both direct effects (context stability on automaticity and automaticity on HRGA) and the total effect as well as indirect effect (context stability on HRGA) on Level 1 and on Level 2. The expected context stability effects emerged even in a fuzzy, natural, real-life setting with people from all over the world with a balanced gender ratio, who intentionally build new habits of many different kinds with minimal, standardized, and automatic guidance by the app software in a self-regulated manner without direct incentives like course credit. Thus, Study 2 provides an indication for generalizability of the context effects in habit building we proposed in our hypotheses.

## General Discussion

The goal of this paper was to test how context stability affects the development of new, beneficial habits. Both studies provide evidence for the hypotheses that context stability improves automatization and enhances habit performance as indicated by higher HRGA. With the manipulation of context stability by having participants constantly switch places and times in Study 1, it could be shown that these effects vanished if context stability only fluctuates on a generally very low level. Only when participants were instructed to keep their contexts stable (same place and same time), which was the case for the stable context group in Study 1 and all app users of Study 2, the context effects emerged. When people performed their habits in more stable contexts, they reported higher automaticity scores and attained higher degrees of their set habit repetition goals. The effect of context stability on HRGA was partially mediated by automaticity in both studies.

Context seems to be more than just a cue for habit instigation. Here, we observed a positive lingering effect of performing one’s habit in the “correct” context, meaning in the context in which the habit behavior has been encoded in previous repetitions.

### Lingering Context Effects

Analogous to the distinction Gardner et al. (2016) make between instigation automaticity and execution automaticity, it is worthwhile to distinguish two kinds of context effects: Trigger effects and lingering effects. This paper focuses specifically on in habit research scarcely investigated lingering context effects as all measurement points stem from performed habit repetitions: What is the ongoing impact of context on the execution of self-regulated behavior? Both types of context effects have different mechanics in influencing habits, especially in the way they activate. Trigger effects are likely to have a threshold-avalanche activation in the sense that if a context has acquired the associative strength to activate the thought of a habit, then it will be executed given a sufficient instigation automaticity. Lingering context effects should have a more linear influence on habit execution. It is easier to think of it as a tax on execution automaticity that gets higher the less stable the context is perceived up until a threshold at which it differs so much from the usual habit context that it does not matter anymore. Thus, a habit with high execution automaticity should more easily be transferrable to different contexts because of a higher automaticity buffer, while fragile habits (e.g., newly developing and/or complex ones) would suffer much more even from slightly degraded context conditions by taxing already scarce executional automaticity, which would result, as we know, in higher motivational impairments during the habit performance (Stojanovic et al., 2020, 2021). As modern habit research evolves, the resolution of our understanding goes from rather coarse to more refined: From mere frequency to automaticity (Gardner, 2012) to differentiated instigation and execution automaticity (Gardner et al., 2016); and from mere stable contexts (e.g., Ouellette and Wood, 1998) to measured perceived context stability (this paper) to potentially differentiated trigger and execution effects of context in the future. However, context as a part of habit anatomy is currently still pixelated and more research is needed to empirically distinguish these two types of assumed context effects.

### Perception of Context

We know that one should keep the context stable when building new habits. We defined context as a combination of the physical environment and the time in the flow of the day and measured the reported stability which was perceived by the participants. But what are the defining factors that have to be perceived to trigger the perception of context? Is daylight as a zeitgeber more important than the preceding action of just having finished lunch? Is more information derived from visual information about the physical environment than from other variables like temperature? Does the impact of such context information vary systematically over persons, is it idiosyncratic or habit-dependent? There is evidence that different features of context such as the physical location, time of day, mood, and the presence of particular other people can be more or less important for guiding habitual behaviors of different kinds (e.g., purchasing fast food, watching TV news), albeit with physical location having a dominating effect (Ji and Wood, 2007). The results of the studies presented in this paper suggest that the physical environment and the time of day play a central role in context stability perception. The context variation of Study 1, which was induced by having to switch places and times for habit performance, greatly reduced the reported context stability. However, even with this manipulation, the variable context group still reported some context stability - not zero. This might be due to generalized context factors. Even after having switched from the living room to the kitchen for the following habit repetition, a more general representation of the context would be “at home” and different times in the flow of a day could be classified in more general terms like “after school” or “when it is bright outside.” The question of how much the context term can be bended by further generalization before all similarity to the internal reference context is gone, remains open for further research. However, Study 1 gives us a first hunch in so far that the distance on a 11-point scale between around 3 (variable context group) to around 8 (stable context group) was sufficient to change underlying mechanics influencing automaticity and goal attainment in self-regulated behavior. Also, more research on the relative impact of certain classes of context factors for the perception of context stability could yield valuable insights for habit research and improve related interventions.

### Motivational and Habitual Control

When intentionally building good habits with specifically defined goals, motivation and intention are necessary in the beginning to build automaticity. Verplanken and Orbell (2022) describe how attitudes can be the starting point of habit formation by providing the initial motivation to pursue a not yet automized behavior. If the behavior is then repeated in stable contexts over time, control gradually shifts to automaticity, which is accompanied by an increasing resistance to outcome devaluation (Neal et al., 2011; Lattarulo et al., 2019). We show in this paper that an instable context reduces goal attainment—an important outcome related feedback on one’s performance. The degree of goal attainment as an outcome indicator should then be less important for more automized behavior. Hence, varying one’s context could lead to impaired motivation and even cause habit discontinuity, especially at the beginning of the habit forming process if there is not enough automaticity buffer to reduce the impact. But also if the desired behavior is already mainly controlled by habit, context is key for triggering the behavior. So, either way, context—and its stability—impacts the probability of desired behavior being executed both in the realm of motivational as well as in the realm of habitual control. However, longitudinal studies on automaticity-dependent effects of performance feedback in intentional habit building are needed to further support this rationale.

### Implications

To better automize a beneficial habit, make sure to keep the context stable. Here are two important remarks on this.

First, make sure to define context precisely. As we saw in Study 1, it can already heavily disrupt perceived context stability if one just defines a very general context such as “at home after school.” Defining the exact place and the preceding action in the flow of the day seem to be viable heuristics to significantly increase context stability and thus improve habit development and goal attainment.

Secondly, consider the inherent natural variability of the chosen context. For example, a café might vary regularly in how crowded it is, which table one gets on a given day, what background music is playing and whether the adjacent table is occupied by a loudly laughing young couple or a stressed but silent PhD student. The kitchen table at home might have a lower contextual variance and would thus bear less potential to disrupt habit execution. Superficially, both contexts—“at my kitchen table” and “at my favorite café”—seem to be equivalently suitable for habit building, but the difference in their context variance makes one superior to the other. This reasoning can analogously be applied to the time dimension of context. The context variance might be systematically lower in the morning and the evening, but vary more heavily in the middle of the day.

### Limitations

Even though the data from two distinct datasets converged into the same, expected pattern, one should be careful before generalizing the results of this paper to other domains with different conditions. Both datasets consist of mainly young people who intended to build a useful habit. In Study 1, most participants were extrinsically incentivized by course credit and a lottery for an online coupon. Study 2 was constrained to iPhone users. All analyzed habits were defined in a similar way following the structured habit definition process. Hence, all habits share certain attributes that other habits might not have and which might influence the applicability of the presented results. For example, all habits were planned out in advance, should by definition only be performed once a day and were connected with individual long-term goals. Habits of other archetypes, such as smartphone checking habits that get triggered about every 12 min (Ofcom, 2018), or mental habits with less clear cueing mechanisms (Colvin et al., 2021) might differ in their relation to physical and time of day related context. Further, as these studies tracked developing new habits, one cannot exclude that the influence of context could change in very automized habits with high repetition counts. Very strong habits might be less prone to friction in habit execution by variable contexts and could thus more easily migrate to new contexts.

## Conclusion

In this paper, we quantified the influence of context stability in the process of habit acquisition. Keeping the context stable supports behavioral automatization, i.e., habit development. The presented evidence suggests that context does not only act as a trigger to initiate habits, but has a lingering effect with context stability being conductive to a smooth habit execution with higher goal attainment. Having a clear specification of time and space of a habit that is to be built seems to be a good starting point to ensure context stability.

## Data Availability Statement

The raw data supporting the conclusions of this article will be made available by the authors, without undue reservation.

## Ethics Statement

The studies involving human participants were reviewed and approved by Ethik-Kommission der Universität Bielefeld. Written informed consent for participation was not required for this study in accordance with the national legislation and the institutional requirements.

## Author Contributions

MS developed the idea and study design, created the iPhone application, conducted the data acquisition, performed the statistical analyses, and wrote the manuscript. AG and SF contributed to the final version of the manuscript and provided critical feedback, which helped shaping the theoretical foundation of the paper. SF supervised the project. All authors contributed to the article and approved the submitted version.

## Conflict of Interest

The authors declare that the research was conducted in the absence of any commercial or financial relationships that could be construed as a potential conflict of interest.

## Publisher’s Note

All claims expressed in this article are solely those of the authors and do not necessarily represent those of their affiliated organizations, or those of the publisher, the editors and the reviewers. Any product that may be evaluated in this article, or claim that may be made by its manufacturer, is not guaranteed or endorsed by the publisher.

## Footnotes

^1^ https://www.qualtrics.com

^2^The demographic data about app use by country, gender, and age were taken from the google firebase console (the database backend of the app) and also contain the excluded cases with less than two habit repetitions and data from the first author as it was technically not possible to exclude specific cases from this analysis.

^3^The SRHI-items 2–11 were used. However, items 4, 6, and 11 were removed with one app update. Thus, 4 and 6 only had 39 measurement points each and item 11 had 110, while the rest of the items had contributed each between 727 and 834 measurement points. The average values of the automaticity scale with and without items 4, 6, and 11 correlated with *r* = 0.99, which is why we decided to keep the underrepresented items in the scale and not lose the data points.
